# Metabolic characterization of directly reprogrammed renal tubular epithelial cells (iRECs)

**DOI:** 10.1038/s41598-018-22073-7

**Published:** 2018-03-01

**Authors:** Simon Lagies, Roman Pichler, Michael M. Kaminski, Manuel Schlimpert, Gerd Walz, Soeren S. Lienkamp, Bernd Kammerer

**Affiliations:** 1 Center for Biosystems Analysis (ZBSA), Albert-Ludwigs-University Freiburg, Habsburgerstr. 49, 79104 Freiburg, Germany; 2 Spemann Graduate School of Biology and Medicine (SGBM), University of Freiburg, Albertstr. 19a, 79104 Freiburg, Germany; 3 Faculty of Biology, University of Freiburg, Schänzlestr. 1, 79104 Freiburg, Germany; 4 Department of Medicine, Renal Division, Medical Center—University of Freiburg, Faculty of Medicine, University of Freiburg, Hugstetter Str. 55, 79106 Freiburg, Germany; 5 BIOSS Centre of Biological Signalling Studies, University of Freiburg, Schänzlestr. 18, 79104 Freiburg, Germany

## Abstract

Fibroblasts can be directly reprogrammed to induced renal tubular epithelial cells (iRECs) using four transcription factors. These engineered cells may be used for disease modeling, cell replacement therapy or drug and toxicity testing. Direct reprogramming induces drastic changes in the transcriptional landscape, protein expression, morphological and functional properties of cells. However, how the metabolome is changed by reprogramming and to what degree it resembles the target cell type remains unknown. Using untargeted gas chromatography-mass spectrometry (GC-MS) and targeted liquid chromatography-MS, we characterized the metabolome of mouse embryonic fibroblasts (MEFs), iRECs, mIMCD-3 cells, and whole kidneys. Metabolic fingerprinting can distinguish each cell type reliably, revealing iRECs are most similar to mIMCD-3 cells and clearly separate from MEFs used for reprogramming. Treatment with the cytotoxic drug cisplatin induced typical changes in the metabolic profile of iRECs commonly occurring in acute renal injury. Interestingly, metabolites in the medium of iRECs, but not of mIMCD-3 cells or fibroblast could distinguish treated and non-treated cells by cluster analysis. In conclusion, direct reprogramming of fibroblasts into renal tubular epithelial cells strongly influences the metabolome of engineered cells, suggesting that metabolic profiling may aid in establishing iRECs as *in vitro* models for nephrotoxicity testing in the future.

## Introduction

The growing incidence of chronic kidney disease leads to various socio-economic implications and represents a major challenge for health care systems worldwide^1^. There is an unmet demand for new *in vitro* models of kidney diseases to develop new diagnostic and therapeutic methods and to get a better insight into molecular mechanisms of kidney diseases. In the last decade, enormous progress has been made in generating kidney cells *in vitro*, including the *in vitro* expansion of nephron progenitors^2,3^, directed differentiation of induced pluripotent stem cells (iPSCs)^4–7^ and direct reprogramming^8,9^. These approaches have the potential to circumvent some of the disadvantages of primary kidney cells in culture, such as dedifferentiation, limited proliferative capacity and senescence^10–12^. Moreover, newly generated kidney cells resemble their native counterparts and share more characteristics with primary kidney cells than immortalized kidney-derived cell lines like IMCD-3 or HK-2 cells^13^. Therefore, these cells can be established as reliable *in vitro* systems for drug toxicity testing and disease modeling. Furthermore, *in vitro* generated kidney cells could represent a patient-specific source for future cell replacement therapies^5^.

Direct reprogramming is an established approach to convert one cell type into another differentiated cell type bypassing the pluripotent state of iPSCs and the risks associated with this approach. Already accomplished for hepatocytes^14,15^, neurons^16^, cardiomyocytes^17^ and others, we recently managed to directly reprogram fibroblasts to induced renal tubular epithelial cells (iRECs) by forced expression of four transcription factors^8^. By lentiviral transduction of Hnf1β, Hnf4α, Pax8 and Emx2 fibroblasts were converted into iRECs, which exhibit distinct features of differentiated tubular epithelial cells. In contrast to fibroblasts, iRECs express epithelial and tubular surface markers and tubule-specific transporters. Using transcriptional profiling techniques and CellNet^18^- based characterization, we demonstrated that iRECs bear a substantial similarity to primary kidney tubule cells. On an ultra-structural level, they show tight junctions, a clear apico-basal polarity and a basement-membrane like matrix. Importantly, expression of proximal-tubule specific transporters like OCT2 (SLC22A2, organic cation transporter-2) and the apolipoprotein-receptor megalin (LRP2), detection of microvilli and evidence for endocytotic uptake of albumin indicate that iRECs share specific characteristics of proximal tubule cells.

Although iRECs have been analyzed at a morphological and functional level, little is known about metabolic changes that occur in reprogrammed cells. Several studies have dealt with metabolome profiling of induced pluripotent stem cells^19–22^. Bioenergetics analysis of iPSCs revealed that transition from a somatic state to pluripotency was accompanied by a switch from mitochondrial oxidative phosphorylation to glycolytic ATP production^19^. Interestingly, the inhibition of glycolysis prevented iPSC reprogramming. These findings could be confirmed by an independent study using an untargeted metabolomic approach^20^. Comparing iPSCs to human ESCs (embryonic stem cells) and somatic cells (fibroblasts) demonstrated that the metabolic signature of iPSCs resembles that of hESCs^23^. This demonstrates that cellular reprogramming is accompanied by metabolic reprogramming. Recently, the analysis of fully and partially reprogrammed human iPSCs uncovered that the metabolic profile of iPSCs reflected their grade of immaturity^22^. These studies demonstrate that major changes in cell metabolism are not only characteristic of reprogramming, but also play a crucial role in the reprogramming process itself. To our knowledge, no studies have analyzed metabolic features of directly reprogrammed or iPSC-derived kidney cells.

One important application of directly reprogrammed cells could be their use in drug monitoring, toxicity testing of novel compounds and prediction of drug toxicity on a personalized, patient-specific level. We previously demonstrated that iRECs are susceptible to nephrotoxic substances like gentamicin and tacrolimus^8^, showing elevated rates of cell death compared to MEFs and upregulation of Kidney injury molecule 1 (KIM1). Notably, there was also an iREC- specific cytotoxic response to cisplatin (cis-diamminedichloroplatinum II), which could not be detected in MEFs.

Cisplatin is one of the most widely applied chemotherapeutic drugs for the treatment of malignancies like carcinomas and germline tumors. However, like many chemotherapeutic drugs, cisplatin has several relevant side effects, including ototoxicity, myelosuppression, peripheral neuropathy and anaphylaxis^24^. Due to the accumulation in renal tissue, the kidneys are especially susceptible to cisplatin, and nephrotoxicity represents a major, dose-limiting side effect^25,26^. Cisplatin-mediated toxic damage often leads to acute kidney injury (AKI), induced by proximal tubular injury, oxidative stress, vascular injury and inflammation^24^. The S3 segment of the renal proximal tubule, which is mainly located in the kidney medulla, is particularly sensitive to nephrotoxic substances^27^. Uptake of cisplatin is mediated by organic cation transporter-2 (OCT2), on the basolateral side of proximal tubule cells^28^. OCT2 is also expressed in iRECs^8^. After uptake, cisplatin is converted into reactive metabolites^29^. By forming DNA intra- and interstrand cross-links^30^, it mainly interferes with DNA synthesis and targets rapidly proliferating cells. Following cisplatin- induced DNA damage and DNA damage repair (DDR), multiple molecular pathways are activated, such as p53 signaling^31^, apoptosis pathways, autophagy^32^ and TNFα expression^33^. These processes, including mitochondrial dysfunction and activation of the mitochondrial apoptotic pathway^34^, have various implications for the transcriptome, proteome and metabolome of affected cells^35^.

Consequently, several studies aimed at analyzing cisplatin-induced metabolic alterations in cells treated with cisplatin^27,35–41^. There is a known depletion of amino acids in tubular cells after cisplatin application, mainly caused by elevated levels of aminoaciduria and deteriorated reabsorption of amino acids^27,36,37^. Notably, branched-chain amino acids like leucine, isoleucine and valine are elevated in urine and decreased in serum, and could be used as biomarkers to assess individual predisposition for nephrotoxicity as they correlate with common markers for renal function after cisplatin exposure^39^. Moreover, cisplatin impacts lipid metabolism and leads to an accumulation of fatty acids in kidney tissue, due to reduced fatty acid oxidation^35,37,42^. Furthermore, diacylglycerols, triacylglycerols, ceramides and in general neutral lipids accumulate upon cisplatin application^27,43^. In addition to alterations in lipid metabolism, cisplatin has also been shown to reduce enzymes involved in glycolysis^44^. Thus, glucose accumulates by cisplatin application and metabolites involved in glycolysis decrease^45^.

In this study, we analyzed iRECs, mIMCD-3 cells, MEFs and whole kidneys with an untargeted metabolomic approach. Analyzing the endo- and exometabolome by gas chromatography-mass spectrometry (GC-MS), each cell type could be reliably distinguished. iRECs cluster with mIMCD-3 cells but clearly separate from MEFs, indicating that the metabolic profile of iRECs is similar to the profile of mIMCD-3 cells. Treatment with the chemotherapeutic drug cisplatin affected the metabolome of iRECs and mIMCD-3 cells, whereas no changes could be elicited in MEFs. In iRECs and mIMCD-3 cells cisplatin administration led to a decrease of several clusters of metabolites, including amino acids, metabolites involved in glycolysis and the pentose monophosphate pathway, and TCA cycle intermediates. Interestingly, we could detect several well described changes after cisplatin exposure in iRECs, which could not be confirmed in mIMCD-3 cells. In particular, there was an increase of medium- and large-chain fatty acids in the cell and an increase of amino acids in the exometabolome.

## Material and Methods

### Animals

For whole kidney metabolomic analysis kidneys of three adult C57BL/6 N mice were used. After excision with a scalpel blade, kidneys were weighed and directly prepared for further metabolomic analysis. Mice were housed in a SPF facility with free access to chow and water and a 12 h day/night cycle. All animal experiments were conducted according to the National Institutes of Health Guide for the Care and Use of Laboratory Animals, as well as the German law for the welfare of animals, and were approved by the local authorities (Regierungspräsidium Freiburg). MEFs were generated as previously described^8^.

### Cell culture

iRECs were generated by direct reprogramming of mouse embryonic fibroblasts (MEFs) as described previously^8^. In short, KspCre reporter MEFs were transduced with four lentivirus, each containing one of the four transcription factors Hnf1b, Hnf4a, Emx2 and Pax8 cloned into the pWPXLd lentiviral vector (Addgene no. 12258). Virus concentrate was diluted 1:1000 to 1:100 in MEFM containing 8 µg ml^−1^ Polybrene (sc-134220). Cells were incubated for 12 h on 7 consecutive days. 14 days after the last viral transduction GFP-positive cells were sorted using a BD FACSAria^TM^ Fusion flow cytometer (Becton Dickinson). Afterwards, the cells were expanded in MEFM (Dulbecco’s modified Eagle’s medium (DMEM), 2 mM L-glutamine, penicillin/streptomycin, 10% fetal bovine serum (FBS)). All cells were cryoconserved in freezing medium (containing 50% MEFM, 40% FBS, 10% DMSO) and stored in liquid nitrogen.

For metabolomic analysis MEFs, iRECs and mIMCD-3 (ATTC) cells were thawed and counted. 2· 10^6^ cells were seeded per 10 cm culture dish and expanded until harvested. All three cell lines were grown in MEFM containing DMEM High Glucose (4,5 g L^−1^). Medium was changed on a daily basis; the medium for metabolomic analysis was incubated on cells for 24 h before harvesting. For cisplatin exposure, all three cell lines were incubated in MEFM containing 6 µL mL^−1^ cisplatin for 24 h (Cisplatin stock solution 0,1 mg mL^−1^, Apotheke des Universitätsklinikums Freiburg).

### Metabolite extraction

Cells were washed twice on ice with 5 mL 0.9% NaCl. Metabolism was quenched by adding 1.5 mL ice-cold extraction medium, i.e. methanol/water (9/1 v/v) containing 1 µg mL^−1^ ribitol and phenyl-beta-D-glucopyranoside as internal standards. Cells were harvested by scratching them off with cell scrapers and transferred into 2 mL screw-cup vials prefilled with 300 mg glass beads and immediately frozen in liquid nitrogen. Cells were lyzed by a Precellys 24 tissue homogenizer by applying three cycles of 15 s maximal intensity and 10 s break at −20 °C. After centrifugation (21,000 g, 4 °C, 10 min), 550 µL of the supernatant were transferred in a new 2 mL Eppendorf tube and dried overnight in an Eppendorf Concentrator plus vacuum rotator. Metabolite pellets were stored under nitrogen atmosphere until derivatization.

Kidneys were weighed and washed with 0.9% NaCl. The whole kidneys were transferred into screw-cup vials prefilled with 300 mg glass beads and quenched with extraction medium. The volume was adjusted to tissue weight to yield equal concentrations (see supplementary Table S1). Kidneys were lyzed by a Precellys 24 tissue homogenizer by applying three cycles of 30 s maximal intensity and 30 s break at −20 °C twice, with five minutes between the two runs. Lysates were centrifuged (21,000 g, 4 °C, 10 min) and 100 µL were dried in an Eppendorf Concentrator plus vacuum rotator.

Medium was centrifuged (5 min, 20,000 g, 4 °C) to remove detached or dead cells. 100 µL medium were added to 900 µL ice-cold acetonitrile/methanol (3/1 v/v) containing 1 µg mL^−1^ ribitol and phenyl-beta-D-glucopyranoside as internal standards. After 10 s of vortexing, the medium was centrifuged (1 h, 20,000 g, 4 °C) and 100 µL of the supernatant were transferred to a new 2 ml Eppendorf tube and dried for three hours.

### Derivatization

Metabolite pellets gained from cells, tissue or medium were derivatized by methoxymethylation of keto- and aldehyde-groups as well as trimethylsilylation of amines, hydroxyl- and carboxylic groups. Therefor, 20 µL methoxyamine hydrochloride (20 mg mL^−1^ in pyridine) were added to the metabolite pellet and shaken for 90 min at 28 °C with 1200 rpm. Afterwards, 50 µL N-methyl-N-(trimethylsilyl)-trifluoroacetamide were added and incubated for 30 min at 37 °C with 1200 rpm.

### GC/MS-Analysis

1 μL of the derivatized sample was splitlessly injected by a Gerstel MPS2 XL autosampler into an Agilent 7890 A/5975 C system in randomized order (sample order is shown in supplementary Table S2). Chromatographic separation was performed using an HP5-MS column (5%-diphenyl-95%-dimethylpolysiloxane, 60 m x 0.25 mm × 0.25 μm). The gradient was 80 °C hold for 3 min, to 320 °C with 5 °C/min and hold for 14 min. The carrier gas flow rate (He) was set to 1 mL min^−1^ and the septum purge flow was 3 mL min^−1^. Spectra were acquired in full scan mode with a scan rate of 1.99 s^−1^ from 50 m/z to 800 m/z. Equilibration time and post run time was 1 min each. The inlet temperature was 230 °C, the MS source temperature 230 °C and the quadrupole analyzer temperature 150 °C.

### LC/MS-Analysis

Cells or medium pellets were resuspended in 100 µL resuspension buffer (10 mM NH_4_CH_3_CO_2_ pH 9, acetonitrile/water 1/1). 5 µL were injected onto a BEH Amide 150 × 2.1 mm column. The column temperature was set to 40 °C. Two targeted methods were applied: one targeting amino acids, the other one targeting intermediates of glycolysis and the TCA-cycle. Buffer A consisted of 10 mM NH_4_CH_3_CO_2_ pH 9 acetonitrile/water 20/80 and buffer B of 10 mM NH_4_CH_3_CO_2_ pH 9 acetonitrile/water 95/5. The chromatographic program for amino acid separation was 99.9% B for 0.5 min, to 80% B until 0.6 min, to 25% B until 5 min, hold until 8 min, to 99.9% B until 8.5 min and hold until 12 min. Separating intermediates of glycolysis and the TCA-cycle was achieved by the following program: 99.9% B hold for 1 min, to 30% B until 5 min, to 25% B until 5.5 min hold until 8.5 min, to 99.9% B until 9 min and hold until 14 min. Each flow rate was set to 400 µL min^−1^. Multiple-Reaction-Monitoring (MRM) was optimized for each compound with analytical standards using Agilent’s MRM-Optimizer. All transitions are displayed in supplementary Table S3. Instrument parameters (6460 triple quadrupole Agilent Technologies) were set to 350 °C gas temperature, 8 L min^−1^ gas flow, 30 psi nebulizer pressure, 250 °C sheath gas temperature, 5 L min^−1^ sheath gas flow, +3 kV/−3.5 kV capillary voltage and +500 V/−300 V nozzle voltage. As internal standards, ^13^C_3_-pyruvic acid, d_4_-succinic acid and ^13^C_4_-^15^N-aspartic acid were used for intermediates of energy metabolism and amino acids, respectively. The results of methodology validation are displayed in supplementary Table S4.

### Data analysis

Within the GC/MS sample set, there was one C10-C40 n-alkane standard sample for building system independently Kováts’ retention index (RI)^46^.

Automated Mass spectral Deconvolution and Identification System (AMDIS)^47^ was used for retention index calculation, peak identification, deconvolution and integration. A compound matrix was generated by spectconnect online tool^48^ with compounds found in more than 50% of the replicates and an 80% similarity threshold of mass spectra. We applied these rather loose settings purposely in order not to miss too many cell type specific metabolites essential to establish cellular metabolic identity. Compounds were annotated by matching the obtained spectra with library spectra of Nist14^49^, FiehnLib^50^ and golmDB^51^, where a match factor threshold of 750 and a retention index deviation of less than 5% were applied. Annotated compounds were normalized to phenyl-glucose. Further, all compounds were normalized to the sum of all compounds to take different cell numbers and mass into account^52^. For cells, blank plates treated equally as plates containing cells were analyzed the same way and their average intensity of each compound was subtracted from each compound in each condition.

For LC/MS analysis, metabolites were identified by Quantitative Analysis software (Agilent) using retention time and specific MRM-transitions as well as their qualifier ratios. Data were normalized to the isotopically labeled standard according to the corresponding pathway. For cells, averaged blank values were subtracted from each metabolite.

For statistical analysis, metaboanalyst 3.0^53–58^, an R based statistic suit for metabolomics, was used. Half of the minimum positive value of each metabolite replaced missing values in GC/MS analysis. In this study, following tests were performed: ANalysis Of VAriance (ANOVA), Benjamini-Hochberg false discovery rate with a q-value cut-off of 0.05, principal component analysis and heat map generation both including range-scaling (mean-centered and divided by the range of each variable), cluster and distance analysis after Pearson and Ward, pathway analysis as well as Very ImPortant feature (VIP)-plot calculation. A VIP-score above 1 was considered as meaningful.

### Data availability

The dataset with every replicate for endometabolite and exometabolite analysis of this study is shown in supplementary Table S5.

## Results

### Endometabolic profiling of transdifferentiated kidney cells revealed similar fingerprints to immortalized kidney cells and primary kidneys

**Figure 1:**
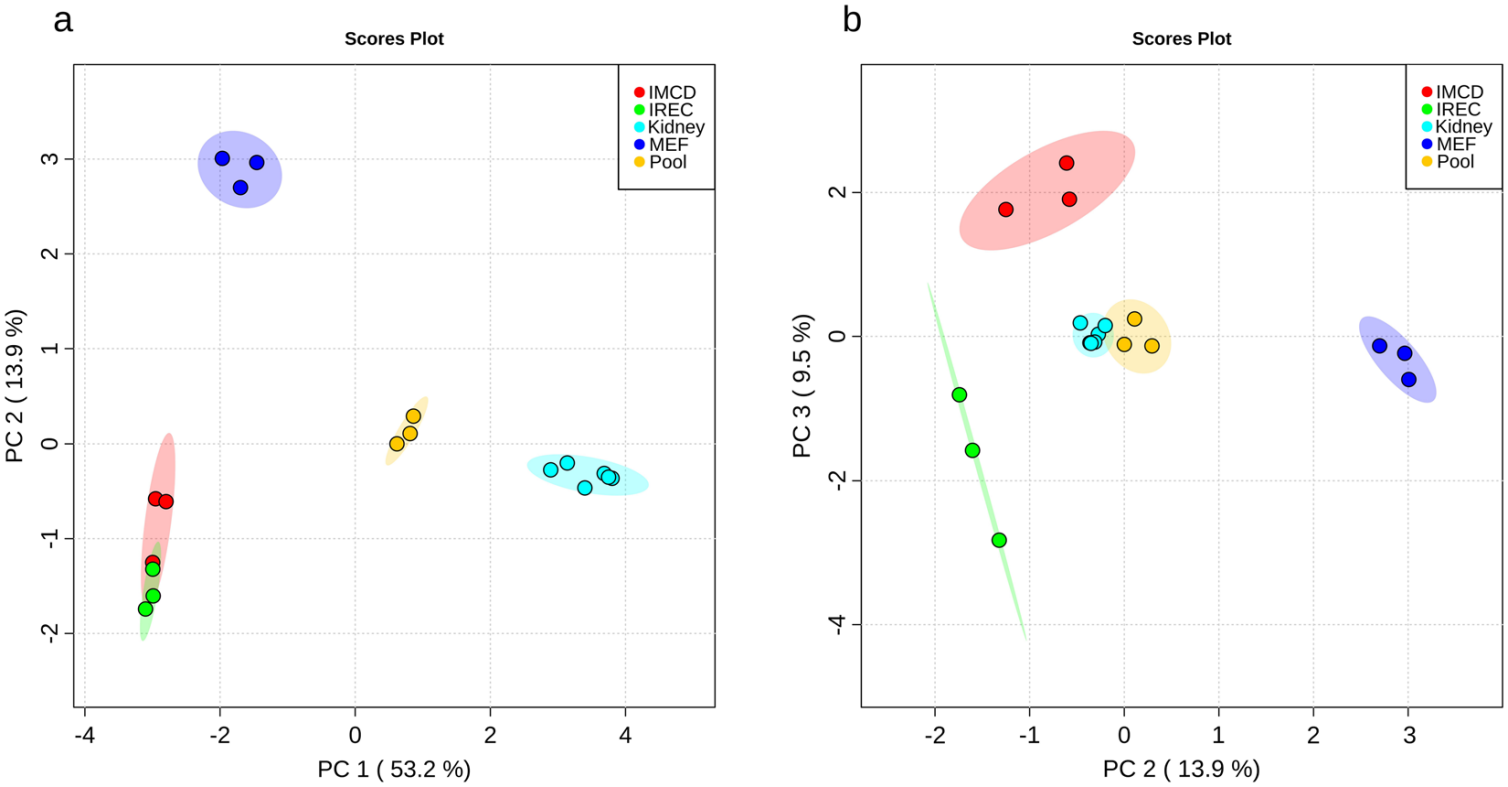
iRECs are similar to other renal epithelial cells and differ significantly from MEFs. (**a**) PCA of endometabolite profiling with PC1 against PC2. (**b**) PCA of endometabolite profiling with PC2 against PC3. blue: MEFs, red: mIMCD-3 cells, green: iRECs, orange: pooled quality control sample, each n = 3; turquoise: whole kidneys, n = 6. Replicates are highlighted as individual dots, shaded area shows the 95%-confidence interval.

To investigate the metabolic identity of iRECs in comparison to other renal epithelial cells and cells they were derived from (MEFs), cultured iRECs, mIMCD-3 cells, MEFs and whole adult kidney lysates were analyzed by untargeted GC/MS based metabolic profiling. Principle component analysis (PCA) revealed that principle component (PC) 1 discriminated between primary renal tissue (positive PC1) and cell culture (negative PC1) with 53.2% of the observed differences (Fig. 1a.) PC 2 accounted for 13.9% of the alterations and distinguished fibroblasts (positive PC2) from kidney cells (negative PC2). Applying two-dimensional PCA, there was no global difference between mIMCD-3 cells and iRECs. Hence, pooled samples serving as quality control were centered between the four groups and had low intra-group variance. In PC3, iRECs and mIMCD-3 cells were separated with 9.5% of all differences occurred (Fig. 1b).

**Figure 2:**
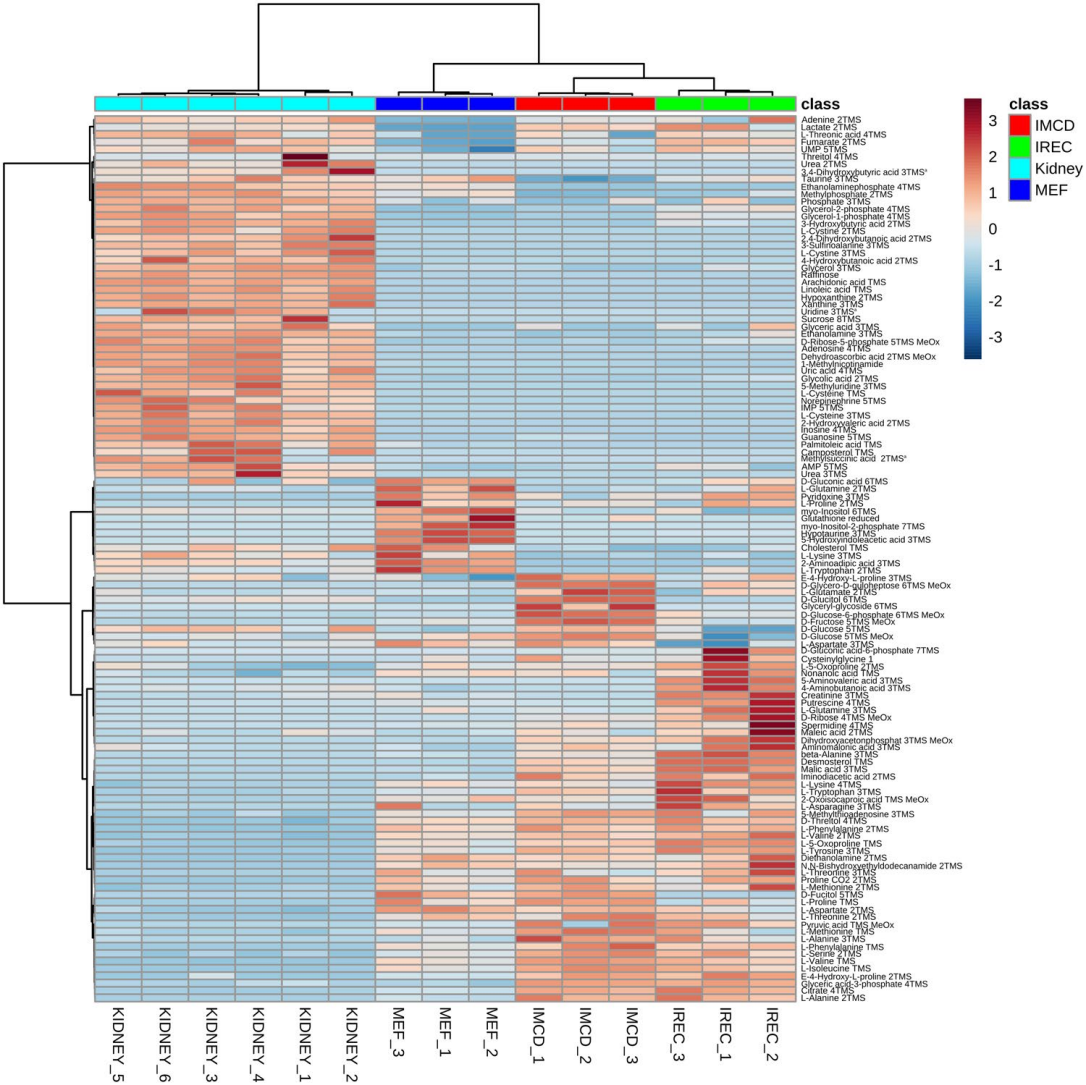
Heat map and cluster analysis of endometabolites reveal similarity between renal epithelial cells. Range-scaled Z-scores of annotated features with a q-value < 0.05 according to ANOVA and FDR correction. Differently abundant metabolite clusters after Pearson and Ward reflect the similarity between cultured renal epithelial cells and iRECs in comparison to MEFs. Marked differences are observable between cultured cells and primary tissue. blue: MEFs, red: mIMCD-3 cells, green: iRECs, each n = 3; turquoise: whole kidneys, n = 6. ^a^: not significant after Tukey’s HSC post-hoc analysis.

Figure 2 shows a heat map of metabolites with an FDR-corrected q-value < 0.05 (all ANOVA and Tukey’s HSD results are shown in supplementary Table S6). The main differences were observed between kidney lysates and cultured cells as shown by Ward clustering. These alterations are also representing the discrimination of kidney tissue to cell culture samples by PC1 (Fig. 1a). The contributing metabolites were products or intermediates of organismal catabolism like urea, 3-sulfinoalanine or modified nucleosides as well as typical animal fatty acids like arachidonic acid, linoleic acid or palmitoleic acid which are either uniquely present or higher abundant in kidney tissue than in cultured cells. Additionally, the metabolic extract was higher concentrated using kidneys compared to cells obtained from culture and thus more metabolites were detected in kidneys, which were below the detection limit in cell culture. This group mainly consisted of nucleosides and nucleobases.

The heat map also shows the different clusters distinguishing cell types. Here, the differences were not based on cell type specific metabolites but different concentration ratios and included mainly amino acids, sugars and organic acids and their related metabolites. In MEF cells, metabolites involved in sugar and energy metabolism were in general strikingly different to mIMCD-3 cells as well as iRECs. These clusters consist, among others, of glyceric acid-3-phosphate, lactate, citrate and fumarate. This result might reflect the high energy demand of kidney cells. Interestingly, iRECs and mIMCD3 cells are mainly discriminated by increased concentrations of biogenic amines such as beta-alanine, putrescine or spermidine in iRECs as well as increased concentrations of sugars in mIMCD-3 cells.

### Exometabolic profiles showed clustering between mIMCD-3 cells and iRECs but not MEFs

**Figure 3:**
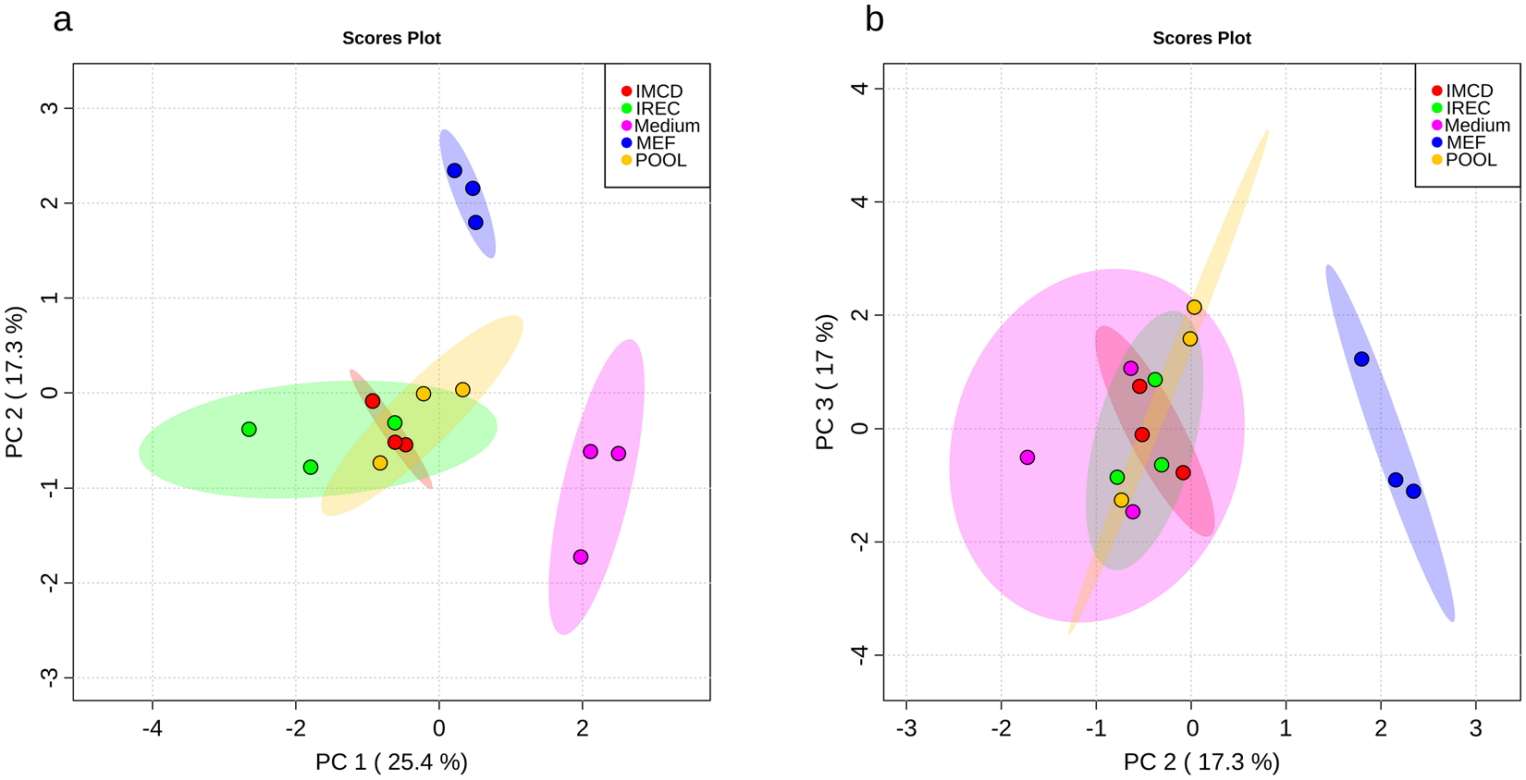
Exometabolites from iRECs are similar to those from mIMCD-3 cell medium and not to MEF medium. (**a**) PCA of exometabolite profiling with PC1 against PC2. (**b**) PCA of exometabolite profiling with PC2 against PC3. blue: MEFs, red: mIMCD-3 cells, green: iRECs, pink: blank medium, orange: pooled quality control sample, each n = 3. Replicates are represented by individual dots, shaded area shows the 95%-confidence interval.

**Figure 4:**
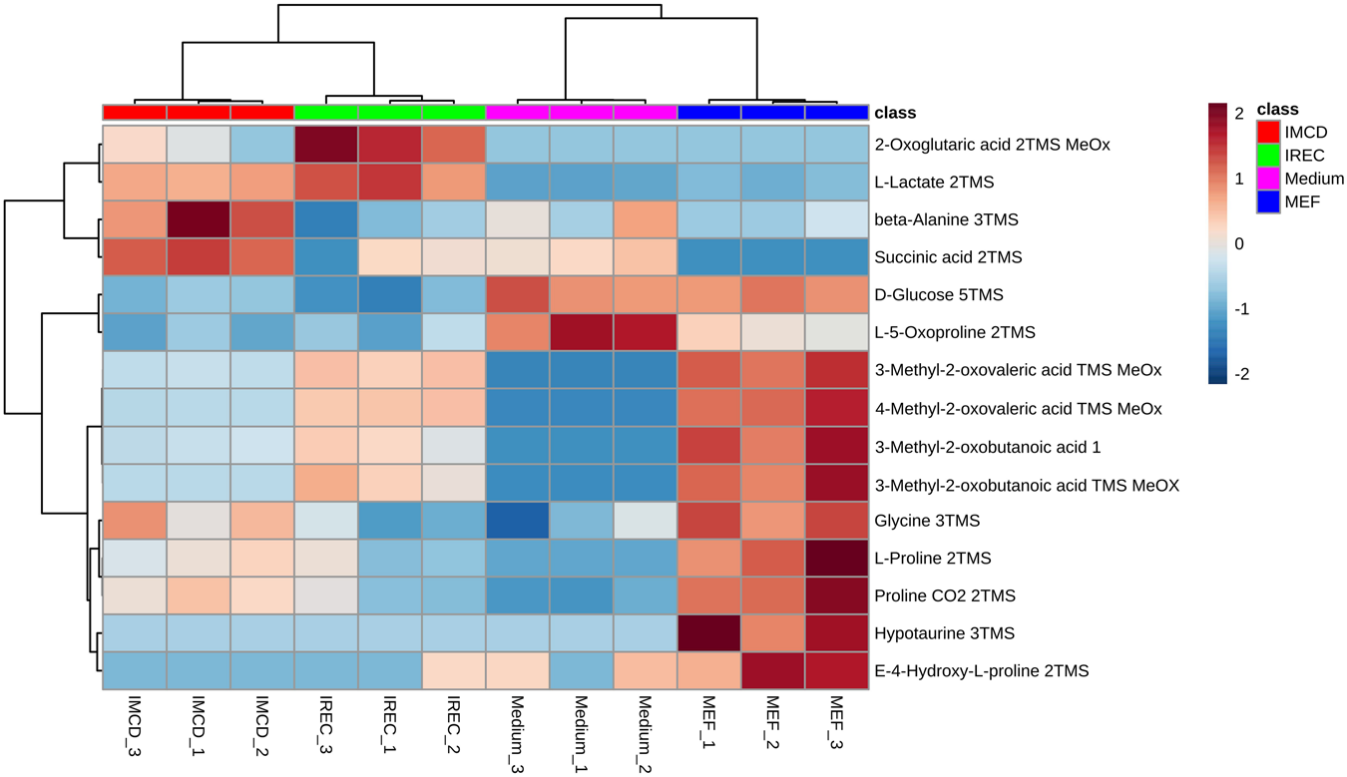
Heat map and cluster analysis of exometabolome. Range-scaled Z-scores of annotated features with q-value < 0.05 according to ANOVA and FDR. Differently abundant metabolite clusters after Pearson and Ward reflect the similarity between mIMCD-3 cells and iRECs in comparison to MEFs. blue: MEFs, red: mIMCD-3 cells, green: iRECs, pink: blank medium, each n = 3.

To explore whether mIMCD-3 cells and iRECs also behave similarly in their excreted metabolites, cell culture medium was analyzed. Due to the analysis of cell culture medium, no data is available for the exometabolome of whole kidneys. PCA revealed discrimination between cell- conditioned and unused medium in PC1 as expected (Fig. 3a). PC2 showed a significant difference between cultured kidney cells and fibroblasts. Hence, we could not detect a difference between iRECs and mIMCD-3 cells in any PC dimension (Fig. 3). Figure 4 shows the corresponding heat map with significantly altered metabolites according to FDR-corrected q-values from ANOVA. Unused medium was not considered in the ANOVA to highlight only those metabolites which actually differed between the cell types. Two clusters lead to the difference between MEFs and kidney cells: the first one is composed of glucose and amino acids and their relatives which were higher in MEF medium. The second cluster consists of glycolysis and TCA-cycle intermediates which were decreased in MEF medium. Nevertheless, some differences between mIMCD-3 cells and iRECs regarding the amino acids and their derivatives, succinic acid and 2-oxoglutaric acid were detected.

### Endometabolome changed upon Cisplatin treatment in iRECs and mIMCD-3 cells but not in MEFs

**Figure 5:**
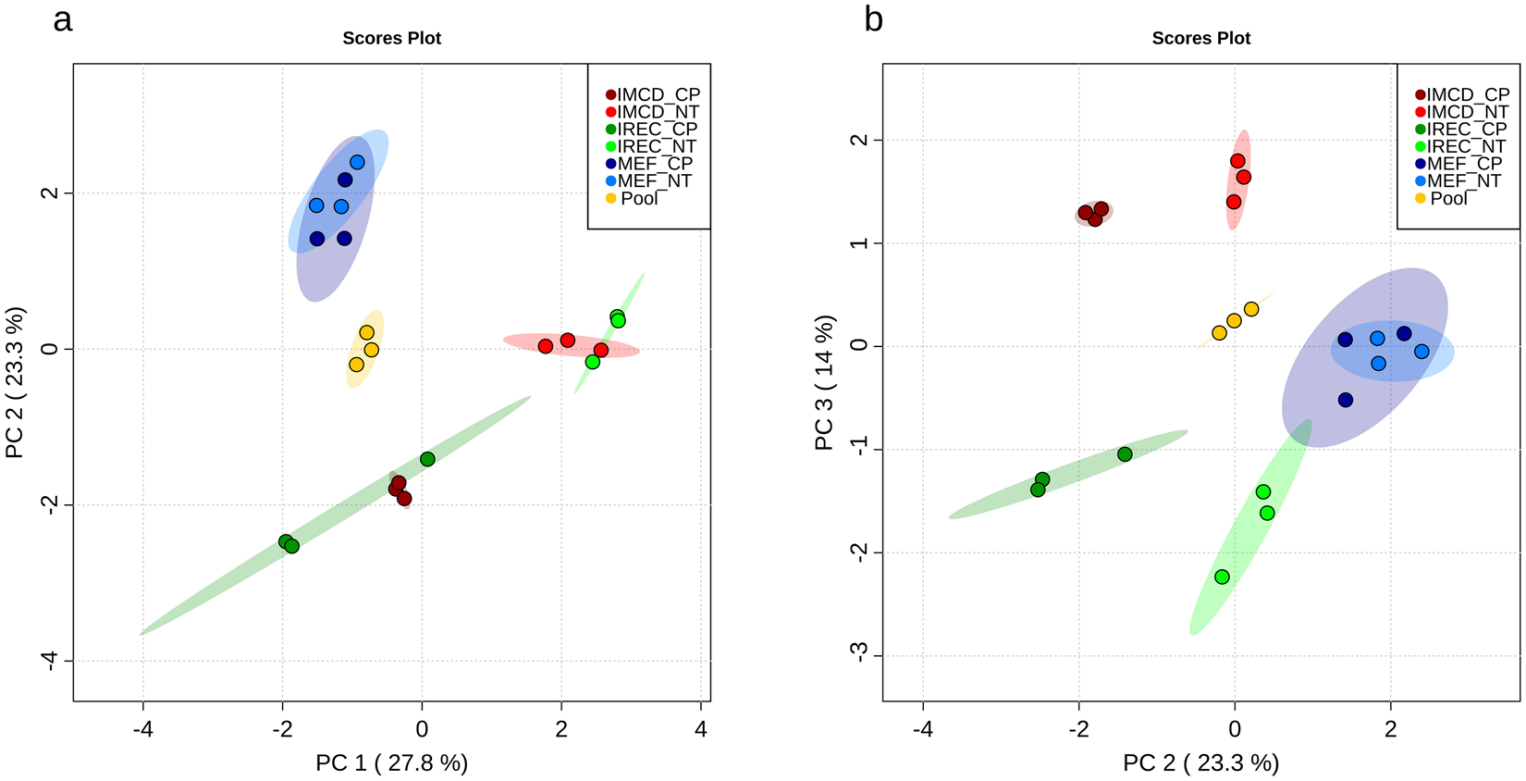
Cisplatin affects the endometabolome exclusively in renal epithelial cells and iRECs. (**a**) PCA of endometabolite profiling with PC1 against PC2. (**b**) PCA of endometabolite profiling with PC2 against PC3. light color: untreated, dark color: cisplatin-treated; blue: MEFs, red: mIMCD-3 cells, green: iRECs, orange: pooled quality control sample, each n = 3. Each dot represents a replicate, shaded area shows the 95%-confidence interval.

To further test the suitability of iRECs as an *in vitro* model for renal epithelial cells, we tested the impact of cisplatin on MEFs, mIMCD-3 cells and iRECs. Due to its long use as a chemotherapeutic agent and its dose limiting nephrotoxicity, cisplatin is well studied in *in vivo* animal models and metabolically well characterized^27,35–40^. As can be seen in the PCA, cisplatin had no global effect on MEFs, neither in PC1, PC2 nor PC3 (Fig. 5). However, cisplatin induced metabolic alterations in kidney cells which could be observed in PC1 as well as in PC2 (Fig. 5a). PC3 did not discriminate between cisplatin treated and non-treated cells but iRECs and mIMCD-3 cells (Fig. 5b).

**Figure 6:**
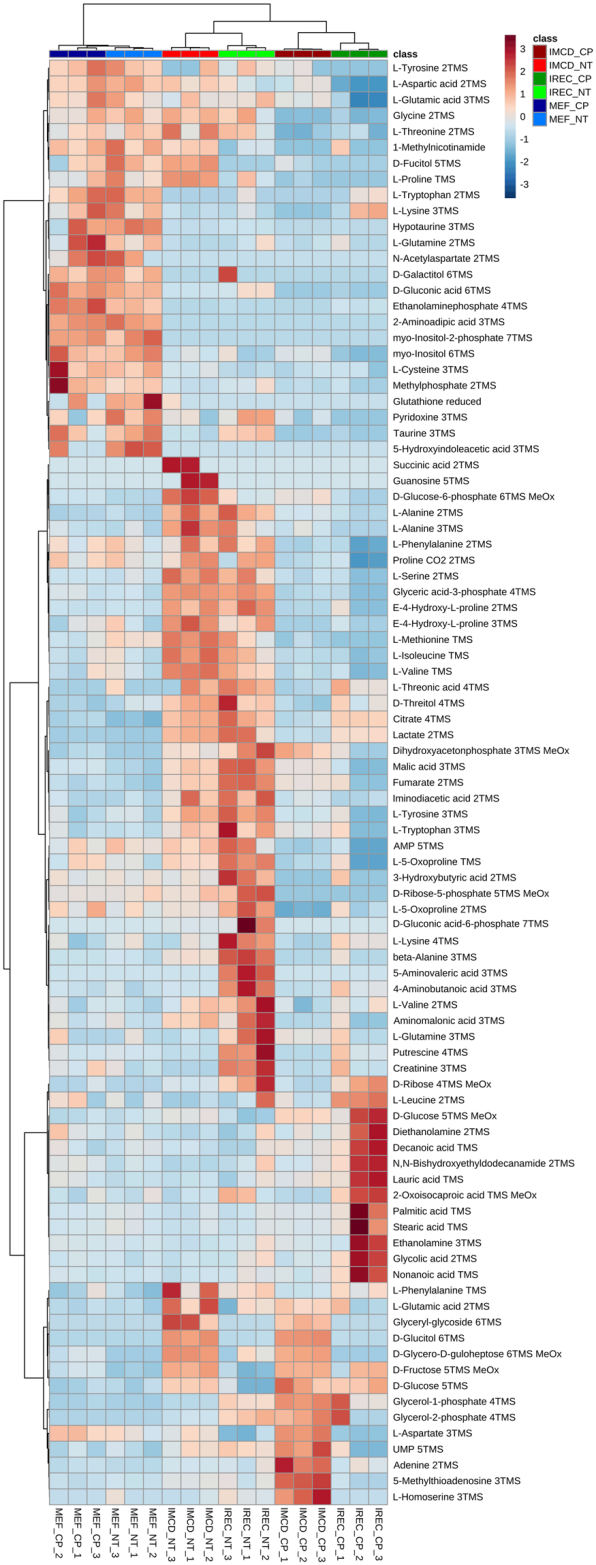
Heat map and cluster analysis of endometabolome discriminates cisplatin treatment only in renal epithelial cells. Range-scaled Z-scores of annotated features with a q-value < 0.05 according to ANOVA and FDR. No effect could be observed in MEFs after cisplatin application, while there was a strong influence on renal epithelial cells. Amino acids and intermediates of glycolysis, TCA-cycle and pentose phosphate pathway were concomitantly down regulated by cisplatin in iRECs and mIMCD-3 cells, whereas fatty acid accumulation was unique to iRECs. light color: untreated, dark color: Cisplatin-treated; blue: MEFs, red: mIMCD-3 cells, green: iRECs, each n = 3

The heat map shows significantly altered metabolites of the endometabolome (Fig. 6). In MEFs, the two main clusters of either highly abundant or lowly abundant metabolites did not change after cisplatin treatment. In contrast, the main clusters of renal epithelial cells changed in response to cisplatin. After cisplatin treatment, a concomitant decrease in many metabolites was observed including many amino acids as well as glycolysis, pentose monophosphate pathway and TCA-cycle intermediates. One cluster showed an increase of metabolites after cisplatin treatment that was only observed in iRECs. Contributing features were medium-chain and large-chain fatty acids, as well as glucose. At the bottom of the heat map, minor clusters appeared which showed differences between mIMCD-3 cells and iRECs independently of cisplatin, and an increase of metabolites induced by cisplatin specifically in mIMCD-3 cells. To determine which pathways were particularly affected, we performed a pathway analysis comparing the specific impact of cisplatin in the three cell lines. While no significant alteration occurred in MEFs, several pathways were affected in both mIMCD-3 cells and iRECs. This analysis confirmed the results from the heat map as several amino acid pathways, glycolysis, TCA-cycle and some lipid pathways were significantly altered (see supplementary Table S7). As the key alterations changed after cisplatin application could be detected in amino acid and energy metabolism, we performed a targeted LC/MS analysis for amino acids and intermediates of glycolysis and the TCA-cycle. Whereas no significant alteration was observed in MEFs, in both mIMCD-3 cells and iRECs amino acids and energy metabolites were drastically decreased. Glucose accumulation could only be validated in iRECs, although an insignificant trend could also be observed in mIMCD-3 cells (see supplementary Fig. S1 and supplementary Table S6). The clustering confirmed the discrimination between MEFs and kidney cells. Moreover, the impact of cisplatin on the metabolome of renal cells led to a more prominent discrimination than the two renal cell lines did themselves (see supplementary Fig. S1, top). These results are thus consistent with the untargeted GC/MS analysis.

**Figure 7:**
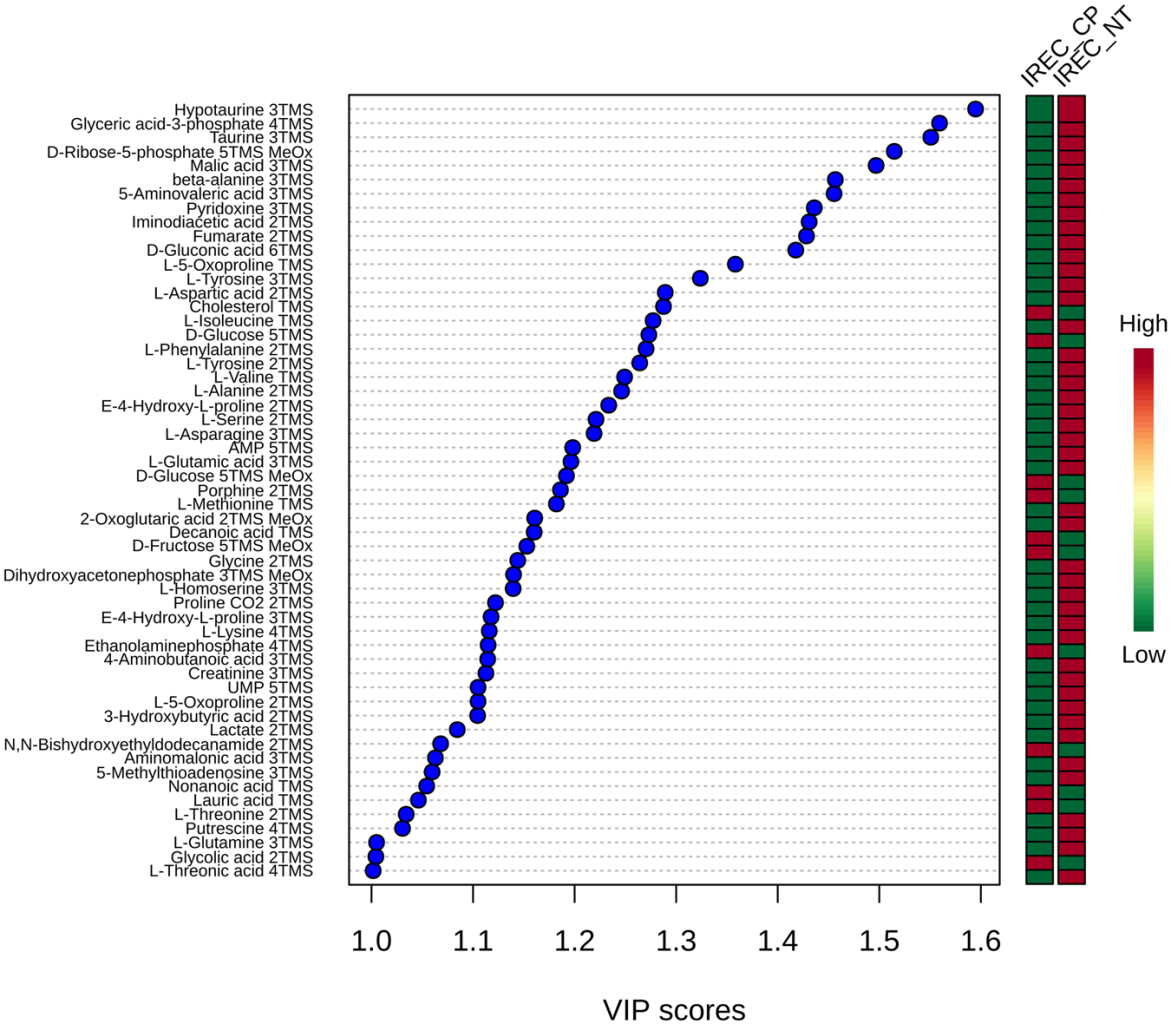
Most important metabolites changed by cisplatin in iRECs. All features with a VIP score > 1 between cisplatin treated and untreated iRECs are displayed. Relative levels are shown for each feature with high abundance (red) and low abundance (green).

To test which metabolites contributed most to the alteration by cisplatin in iRECs, a VIP plot was calculated (detailed results are shown in supplementary Table S8). Figure 7 shows all compounds with a VIP score above 1. The most important features were intermediates from sugar metabolism as well as hypotaurine, taurine, beta-alanine, 5-aminovaleric acid and pyridoxine. Almost all of these metabolites were down regulated.

### Exometabolomics revealed impaired amino acid uptake in iRECs by Cisplatin

**Figure 8:**
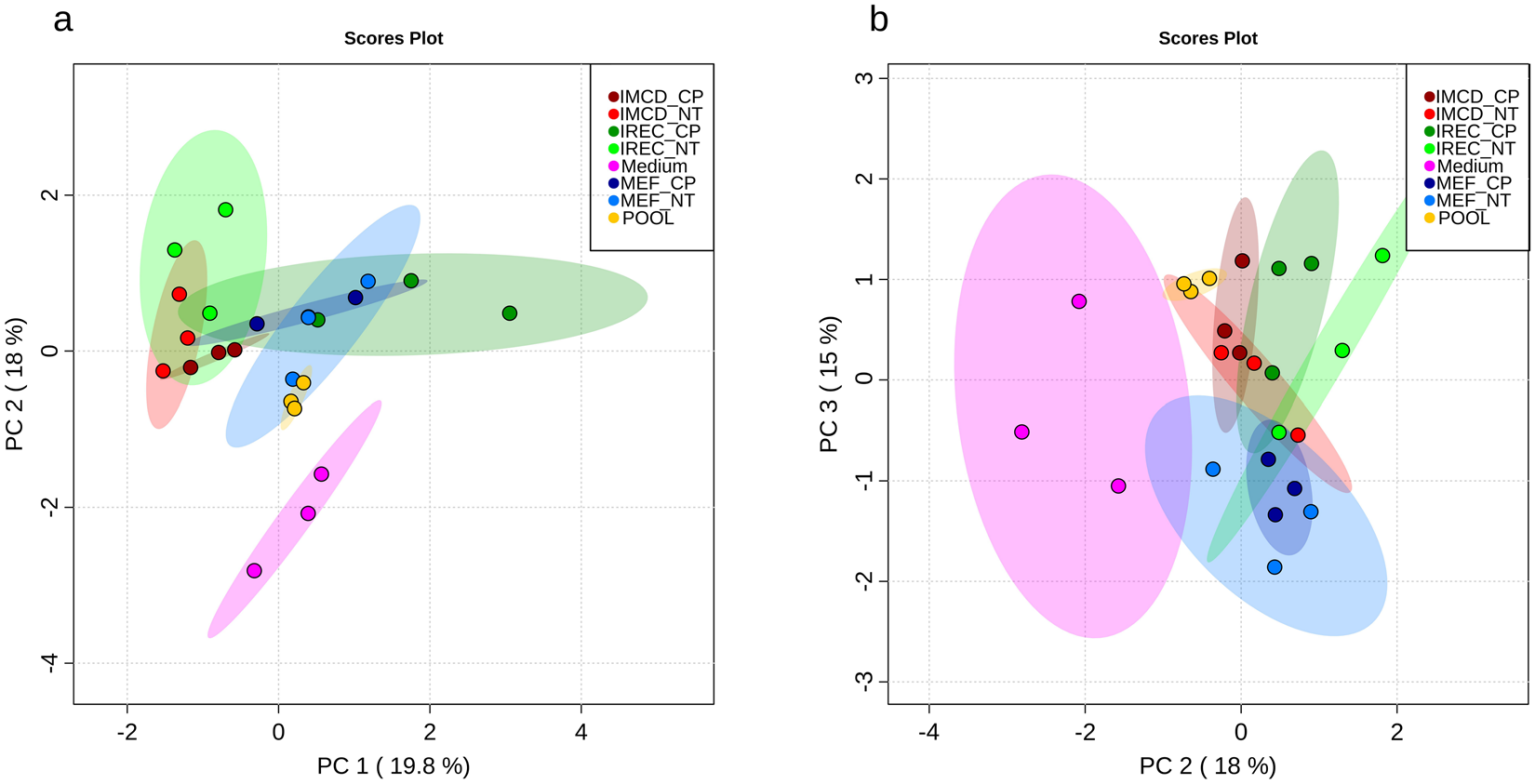
Cisplatin does not influence the exometabolome globally. (**a**) Principal component analysis of exometabolite profiling with PC1 against PC2. (**b**) PCA of exometabolite profiling with PC2 against PC3. light color: untreated, dark color: cisplatin-treated; blue: MEFs, red: mIMCD-3 cells, green: iRECs, orange: pooled quality control sample, each n = 3. Each dot represents a replicate, shaded area shows the 95%-confidence interval.

As shown in Fig. 8, global metabolome analysis could not discriminate cisplatin-treated medium from non-treated medium, neither in PC1, PC2 nor PC3.

**Figure 9:**
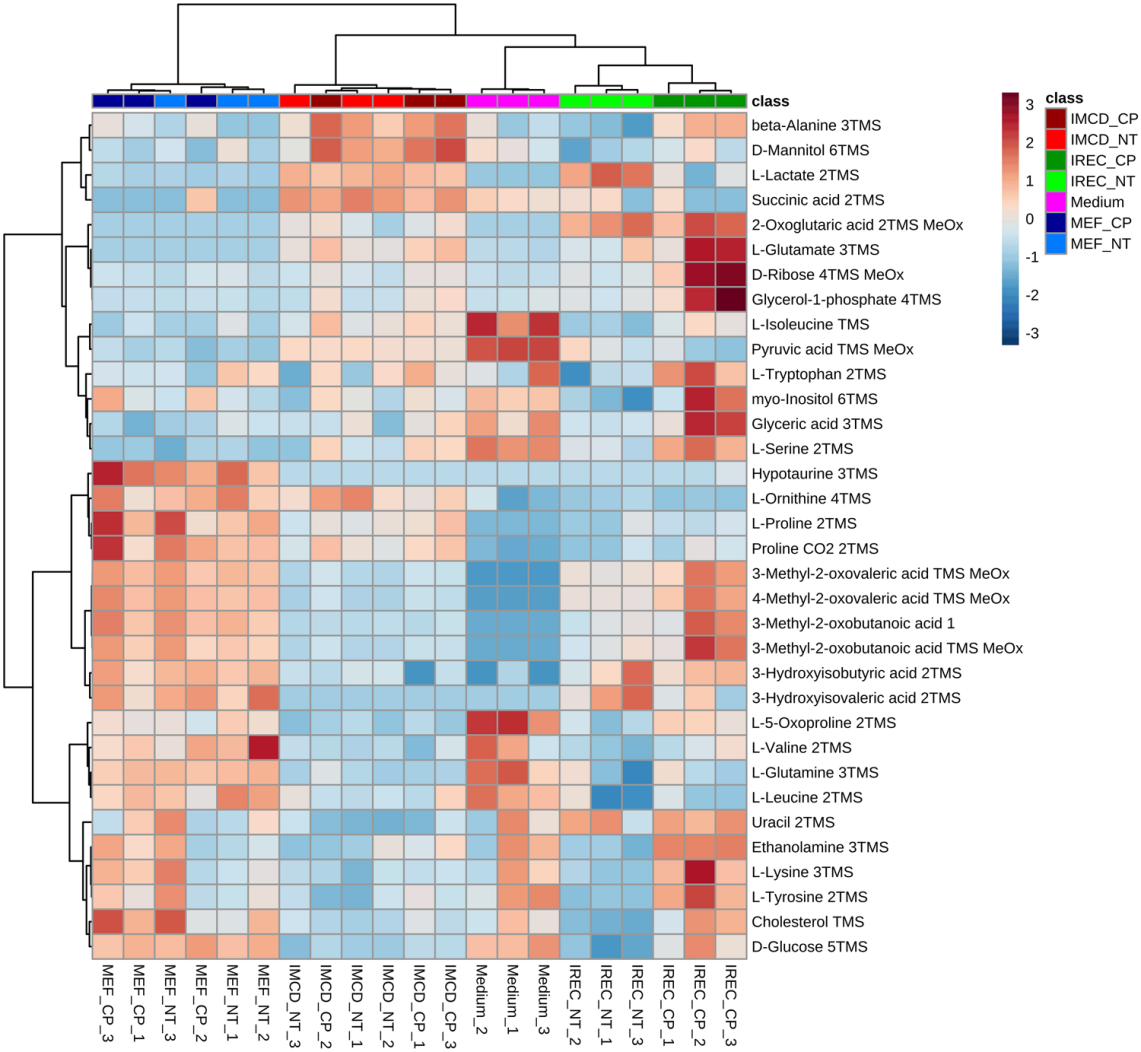
Heat map and cluster analysis of exometabolome after cisplatin-treatment. Range-scaled Z-scores of annotated features with a q-value < 0.05 according to ANOVA and FDR. Medium was excluded for the statistical analysis. No effect could be observed by cisplatin application on MEF and mIMCD-3 medium as there was clustering between both untreated and cisplatin-treated groups. However, clustering differentiated between the medium of treated and non-treated iRECs. Amino acids and their derivatives as well as sugars and lactate were mainly affected. light color: untreated, dark color: cisplatin-treated; blue: MEFs, red: mIMCD-3 cells, green: iRECs, each n = 3.

However, restricting the analysis to ANOVA-positive metabolites revealed differences between treated and non-treated medium from iRECs and no discrimination within MEF medium or mIMCD-3 cell medium (Fig. 9). Contributing metabolites were basically amino acids, which were increased after cisplatin-treatment. Comparison to the unused medium indicated that these increases resulted from both elevated excretion and reduced import. Additionally, there were significant alterations in specific metabolites like sugars, lactate and ethanolamine.

Consistently, targeted validation of the exometabolome by LC/MS led to less prominent results than the targeted endometabolome analysis. No significant alterations were observed in the medium of MEFs and only serine was significantly upregulated in the medium of mIMCD-3 cells. In iRECs medium, serine and cystine were upregulated while glycolysis intermediates pyruvate and lactate were decreased upon cisplatin treatment (see supplementary Fig. S2).

## Discussion

In this study, directly reprogrammed renal epithelial cells were characterized by untargeted metabolomics for the first time. By this approach, we could successfully show that the metabolic profile of iRECs is strikingly similar to that of cultured renal epithelial cells (mIMCD-3 cells). Importantly, cluster analysis could clearly discriminate iRECs from those cells they were derived from (MEFs). The similarity was not only shown by endometabolite profiling but also by analyzing the exometabolome.

To further functionally test the metabolome of iRECs, we applied a well-described nephrotoxic drug, cisplatin, to MEFs, iRECs and mIMCD-3 cells. Global endometabolome analysis revealed significant alterations in the metabolome of mIMCD-3 cells and iRECs upon cisplatin treatment whereas no effects could be observed in MEFs. Strikingly, the alterations were thoroughly in line with published *in vivo* data, including deprivation of amino acids^27,36^ and reduced levels of intermediates of glycolysis, pentose phosphate pathway and citric acid cycle^44,45^. These central findings could be successfully validated by targeted LC/MS analysis of amino acids, glycolysis and TCA-cycle intermediates.

Most interestingly, an accumulation of medium and large chain fatty acids was only observed in iRECs but not in mIMCD-3 cells. These alterations also take place *in vivo* probably by impaired signaling of peroxisome proliferator-activated receptor-α (PPAR-α)^37,59,60^, due to direct cisplatin-mediated inhibition of PPAR-α DNA- binding activity^61^. Consequently, the PPAR-α target gene, mitochondrial medium-chain acyl-CoA dehydrogenase (MCAD), one important enzyme in fatty acid oxidation, was less expressed in cisplatin- treated mice^62^. Consistently, PPAR-α activators lead to elevated MCAD mRNA levels and enzyme activity^62^ and to reduced fatty acid accumulation after cisplatin treatment^37,60^. In kidney tissue and serum of mice treated with cisplatin and the PPAR-α ligand WY, levels of non-esterified fatty acids and triacylglycerols were significantly reduced in contrast to mice treated with cisplatin only. Moreover, treatment with the PPAR-α ligand WY prevented acute renal failure after cisplatin exposure^62^. These observations suggest that the accumulation of fatty acids we observed in iRECs plays a significant pathophysiological role in cisplatin- mediated nephrotoxicity.

In addition, exometabolome analysis revealed an increase in amino acids in the medium of iRECs but neither in mIMCD-3 cells nor MEFs. This is again consistent with aminoaciduria observed *in vivo*^27,36,37^. Targeted LC/MS validation revealed more significant alterations in the medium of iRECs than in the medium of mIMCD-3 cells and MEFs, including amino acids and metabolites of energy metabolism.

Analysis of metabolites most significantly changed upon cisplatin treatment in the endometabolome of iRECs uncovered substances, to which beneficial effects against cisplatin-induced nephrotoxicity have been attributed. These included taurine, pyridoxine and beta-alanine (the latter administered as N-benzoyl-beta-alanine)^63–65^. In summary, our findings suggest that iRECs faithfully reflect many pathophysiological responses to cisplatin. If metabolomic profiling of iRECs represents a good *in vitro* model system for nephrotoxicity testing, will need to be confirmed by testing additional nephrotoxic substances.

The possibility to reprogram human fibroblasts to hiRECs^8^ offers the opportunity to test nephrotoxic agents individually for patients or in a disease specific background. Untargeted metabolomics is not only an excellent technique to test the response of iRECs to new drugs as a risk assessment for potential nephrotoxic side effects, but could also be used to discover agents preventing or attenuating nephrotoxicity of substances in clinical use.

## Electronic supplementary material

Supplementary Tables and Figures S1 and S2

supplementary table S5

supplementary table S6

supplementary table S7

supplementary table S8
