# Supplementary material for: Metabolic characterization of directly reprogrammed renal tubular epithelial cells (iRECs)
Source: Sci Rep. 2018 Mar 1;8:3878. doi: 10.1038/s41598-018-22073-7 (PMC5832874; doi:10.1038/s41598-018-22073-7)
Supplement: Supplementary file 1 — Supplementary Tables and Figures S1 and S2 [file 41598_2018_22073_MOESM1_ESM.pdf]

# Metabolic characterization of directly reprogrammed renal tubular epithelial cells (iRECs) – Supplementary Data

Simon Lagies[1,2,3+], Roman Pichler[4+], Michael M. Kaminski[4], Manuel Schlimpert[1,2,3], Gerd Walz[4,5], Soeren S. Lienkamp[4,5*], Bernd Kammerer[1,5*]

[1] Center for Biosystems Analysis (ZBSA), Albert-Ludwigs-University Freiburg, Habsburgerstr. 49, 79104 Freiburg, Germany

[2] Spemann Graduate School of Biology and Medicine (SGBM), University of Freiburg, Albertstr. 19a, 79104 Freiburg, Germany

[3] Faculty of Biology, University of Freiburg, Schänzlestr. 1, 79104 Freiburg, Germany

[4] Department of Medicine, Renal Division, Medical Center—University of Freiburg, Faculty of Medicine, University of Freiburg, Hugstetter Str. 55, 79106 Freiburg, Germany

[5] BIOSS Centre of Biological Signalling Studies, University of Freiburg, Schänzlestr. 18, 79104 Freiburg, Germany

[+] equal contribution

* Correspondence should be addressed to soeren.lienkamp@uniklinik-freiburg.de or bernd.kammerer@zbsa.uni-freiburg.de

## Supplementary table S1: Adjusted extraction volume for the different tissue weights

| Kidney sample number | Kidney weight [mg] | Adjusted volume for extraction [ml] |
|---|---|---|
| **1** | 240 | 1.50 |
| **2** | 220 | 1.38 |
| **3** | 200 | 1.25 |
| **4** | 210 | 1.31 |
| **5** | 190 | 1.19 |
| **6** | 190 | 1.19 |

## Supplementary table S2: Randomized injection order for untargeted GC/MS profiling and targeted LC/MS validation

| Injection order | GC/MS analysis | LC/MS analysis |
|---|---|---|
| 1 | n-hexane blank | Equilibration pool 1 |
| 2 | C10-C40 alkane standard | Equilibration pool 2 |
| 3 | Equilibration pool 1 | Equilibration pool 3 |
| 4 | Equilibration pool 2 | Equilibration pool 4 |
| 5 | Equilibration pool 3 | Equilibration pool 5 |
| 6 | Equilibration pool 4 | Equilibration pool 6 |
| 7 | Equilibration pool 5 | MEF_CP3 |
| 8 | Equilibration pool 6 | Pool_1 |
| 9 | MEF_NT_3 | MEF_NT_1 |
| 10 | iREC_CP_2 | iREC_CP2 |
| 11 | iREC_CP_3 | IMCD_NT_3 |
| 12 | Kidney_4 | iREC_CP1 |
| 13 | MEF_CP_3 | iREC_NT_2 |
| 14 | Pool_differentiation_1 | iREC_CP3 |
| 15 | iREC_CP_1 | Pool_2 |
| 16 | MEF_CP_1 | MEF_CP1 |
| 17 | Kidney_2 | IMCD_CP1 |
| 18 | Equilibration pool 9 | IMCD_NT_2 |
| 19 | Blank plate_3 | blank1 |
| 20 | MEF_NT_2 | MEF_CP2 |
| 21 | Kidney_3 | iREC_NT_3 |
| 22 | Kidney_6 | IMCD_CP2 |
| 23 | IMCD_NT_3 | MEF_NT_2 |
| 24 | iREC_NT_2 | blank3 |
| 25 | Blank plate_2 | MEF_NT_3 |
| 26 | IMCD_CP_1 | IMCD_NT_1 |
| 27 | Equilibration pool 7 | blank2 |
| 28 | IMCD_NT_2 | iREC_NT_1 |
| 29 | Kidney_1 | Pool_3 |
| 30 | IMCD_CP_3 | IMCD_CP3 |
| 31 | Pool_differentiation_3 | |
| 32 | Kidney_5 | |

| | | |
|---|---|---|
| **33** | iREC_NT_1 | |
| **34** | Pool_CP_1 | |
| **35** | Pool_CP_2 | |
| **36** | MEF_CP_2 | |
| **37** | iREC_NT_3 | |
| **38** | IMCD_CP_2 | |
| **39** | MEF_NT_1 | |
| **40** | Pool_CP_3 | |
| **41** | Equilibration pool 8 | |
| **42** | IMCD_NT_1 | |
| **43** | Pool_differentiation_2 | |
| **44** | Blank plate_1 | |

## Supplementary table S3: Optimized mass spectrometry parameters and retention time of key metabolites changed upon cisplatin

| Compound name | Precursor ion [m/z] | Quantifier/qualifier ions [m/z] | Retention time [min] | Fragmentor Voltage [V] | Collision Energy [V] | Accelerator Voltage [V] | Polarity |
|---|---|---|---|---|---|---|---|
| **$^{13}C_3$-Pyruvate** | 90.1 | 45.1 | 3.18 | 60 | 7 | 3 | Negative |
| **$^{13}C_4{}^{15}N$-Aspartate** | 139.1 | 92.2/77.2, 48.2, 45.2 | 3.88 | 60 | 8/12, 20, 24 | 4 | Positive |
| **3-Phosphoglycerate** | 185 | 97/79, 167 | 5.15 | 60 | 13/37, 5 | 4 | Negative |
| **4-OH-Proline** | 132.1 | 86.1/68.1, 58.2, 41.1 | 3.79 | 80 | 9/21, 25, 33 | 3 | Positive |
| **Alanine** | 90.1 | 44.1 | 3.74 | 45 | 9 | 3 | Positive |
| **alpha-Ketoglutarate** | 145.1 | 101/57.1 | 4.76 | 60 | 4/0 | 4 | Negative |
| **Arginine** | 175.1 | 70.1/130.1, 116.1, 60.1 | 7 | 100 | 25/9, 9, 13 | 3 | Positive |
| **Asparagine** | 133.1 | 74.1/87.1, 46.1, 44.1 | 3.95 | 50 | 13/5, 17, 17 | 3 | Positive |
| **Aspartate** | 134 | 88.1/74.1, 46.1, 43.1 | 3.88 | 50 | 5/9, 13, 21 | 3 | Positive |
| **Citrate** | 191 | 87.1/111.1, 85.1, 67.1 | 5.2 | 60 | 12/8, 12, 24 | 4 | Negative |
| **Cystine** | 241 | 74.2/152.2, 122.2, 120.2 | 4.5 | 84 | 28/8, 16, 20 | 4 | Positive |
| **$d_4$-Succinate** | 121 | 77.1/102.1 | 4.85 | 84 | 9/9 | 4 | Negative |
| **Dihydroxyacetone-phosphate** | 169 | 79/97 | 5.05 | 84 | 25/9 | 4 | Negative |
| **Fructose** | 179.1 | 89.2/113.2, 113.2, 71.2, 59.1, 43.2 | 4.47 | 60 | 4/0, 4, 12, 16, 28 | 4 | Negative |
| **Fructose-1-6-bisphosphate** | 339 | 97/241, 79 | 5.17 | 110 | 13/5, 40 | 3 | Negative |
| **Fructose-6-phosphate** | 259 | 97/169.1, 139, 79 | 5.15 | 84 | 13/9, 13, 49 | 4 | Negative |
| **Fumarate** | 115 | 71 | 4.79 | 60 | 0 | 4 | Negative |
| **Glucose** | 179.1 | 89.1/123.9, 119.1, 71.2, 59.1, 43.3 | 4.72 | 60 | 4/0, 0, 12, 16, 28 | 4 | Negative |
| **Glucose-6-phophate** | 259 | 97/199, 139, 79 | 5.25 | 84 | 13/5, 9, 49 | 4 | Negative |
| **Glutamate** | 148.1 | 84.1/130.1, 102.1, 56.1 | 3.8 | 70 | 13/5, 5, 29 | 3 | Positive |
| **Glutamine** | 147.1 | 130.1, 56.1, 41.1 | 3.9 | 70 | 5/33, 29 | 3 | Positive |
| **Glyceraldehyde-3-phosphate** | 169.1 | 79/151.1, 97 | 5.15 | 80 | 35/15, 15 | 3 | Negative |
| **Glycine** | 76 | 30.3 | 3.9 | 40 | 5 | 3 | Positive |

| | | | | | | | |
|---|---|---|---|---|---|---|---|
| **Histidine** | 156.1 | 110.1/93.1, 83.1, 56.1 | 6.1 | 80 | 9/25, 25, 37 | 3 | Positive |
| **Isocitrate** | 191 | 73.1/173.1, 111, 85.1 | 5.1 | 84 | 21/9, 9, 13 | 4 | Negative |
| **Isoleucine** | 132.1 | 69.1/86.1, 44.2 | 3.15 | 50 | 25/5, 25 | 3 | Positive |
| **Lactate** | 89 | 43.1 | 4.41 | 60 | 15 | 3 | Negative |
| **Leucine** | 132.1 | 43.2/86.1, 44.1, 30.1 | 3 | 60 | 25/5, 21, 13 | 3 | Positive |
| **Lysine** | 147.1 | 130.1/84.1, 67.1, 56.1 | 7.5 | 70 | 5/13, 25, 33 | 3 | Positive |
| **Malate** | 133 | 115.1/73, 71.1, 43.1 | 4.9 | 60 | 4/12, 12, 16 | 4 | Negative |
| **Methionine** | 150.1 | 56.1/133.1, 104.1, 61.1 | 3.2 | 60 | 13/5, 5, 21 | 3 | Positive |
| **o-Tyrosine** | 182.1 | 136.1/165.1, 123.1, 91 | 2.9 | 60 | 9/5, 13, 29 | 3 | Positive |
| **Phenylalanine** | 166.1 | 120.1/103.1, 91.1, 77.1 | 3 | 70 | 9/29, 37, 40 | 3 | Positive |
| **Phosphoenol-pyruvate** | 167 | 79 | 5.12 | 60 | 9 | 4 | Negative |
| **Proline** | 116.1 | 70.1/68.1, 43.1, 28.1 | 3.5 | 70 | 13/33, 33, 37 | 3 | Positive |
| **Pyruvate** | 87 | 43.1 | 3.18 | 60 | 5 | 3 | Negative |
| **Serine** | 106.1 | 60.1/88.1, 70.1, 42.1 | 4 | 50 | 5/5, 9, 5 | 3 | Positive |
| **Succinate** | 117 | 73.1/99.1 | 4.85 | 60 | 8/4 | 3 | Negative |
| **Taurine** | 126 | 44.2/108.1, 97.1, 65.1 | 3.4 | 108 | 20/8, 24, 44 | 4 | Positive |
| **Threonine** | 120.1 | 56.1/102.1, 74.1, 57 | 3.8 | 50 | 13/5, 5, 17 | 3 | Positive |
| **Tryptophan** | 205.1 | 188.1/146.1, 118.1, 91.1 | 2.9 | 70 | 5/13, 25, 40 | 3 | Positive |
| **Tyrosine** | 182.1 | 136.1/165.1, 123.1, 91 | 3.4 | 60 | 9/5, 13, 29 | 3 | Positive |
| **Valine** | 118.1 | 72.1/57.1, 55.1, 29.1 | 3.5 | 50 | 5/29, 17, 29 | 3 | Positive |

## Supplementary table S4: Validation of methodology for key metabolites changed upon cisplatin

| Metabolite | Abbreviation | Sum formula | $R^2$ | Equation of regression | lowest tested concentration with Signal/Noise >= 5 [ng/µl] |
|---|---|---|---|---|---|
| **3-Phosphoglyceric acid** | 3-PG | $C_3H_7O_7P$ | 0.966003 | y = 7.6787x-6.1506 | 15 |
| **4-Hydroxyproline** | 4-OH-Proline | $C_5H_9NO_3$ | 0.996154 | y = 0.3007x-3.1506 | 0.2 |
| **alpha-Ketoglutaric acid** | a-KG | $C_5H_6O_5$ | 0.988594 | y = 0.0037x-0.1506 | 15 |
| **Alanine** | - | $C_3H_7NO_2$ | 0.983956 | y = 0.0747x-0.1506 | 0.0416 |
| **Arginine** | - | $C_6H_{14}N_4O_2$ | 0.843329 | y = 0.0017x-0.1506 | 0.0416 |
| **Asparagine** | - | $C_4H_8N_2O_3$ | 0.997408 | y = 0.0177x-0.1506 | 0.2 |
| **Aspartate** | - | $C_4H_7NO_4$ | 0.99756 | y = 0.0037x-0.1506 | 0.2 |
| **Citrate** | - | $C_6H_8O_7$ | 0.96894 | y = 0.0027x-0.1506 | 15 |
| **Cystine** | - | $C_6H_{12}N_2O_4S_2$ | 0.850668 | y = 0.0017x-0.1506 | 20 |
| **Fructose-6-phosphate** | F-6-P | $C_6H_{13}O_9P$ | 0.985592 | y = 0.0007x-0.1506 | 15 |
| **Fructose** | - | $C_6H_{12}O_6$ | 0.9675 | y = 0.0007x-0.1506 | 15 |
| **Fructose-1,6-bisphosphate** | Fructose-1-6-P2 | $C_6H_{14}O_{12}P_2$ | 0.857609 | y = 3.8027x-8.1506 | 3.12 |
| **Fumarat** | - | $C_4H_4O_4$ | 0.959924 | y = 0.0007x-0.1506 | 0.3 |
| **Glucose-6-phosphate** | G-6-P | $C_6H_{13}O_9P$ | 0.995898 | y = 0.0007x-0.1506 | 6.2 |
| **Glucose** | - | $C_6H_{12}O_6$ | 0.966061 | y = 7.6737x-6.1506 | 0.62 |
| **Glutamate** | - | $C_5H_9NO_4$ | 0.985351 | y = 0.0367x-0.1506 | 0.0416 |
| **Glutamine** | - | $C_5H_{10}N_2O_3$ | 0.994367 | y = 0.5657x-5.1506 | 0.0416 |
| **Glyceraldehyde-3-phosphate** | Glyceraldehyde-3-P | $C_3H_7O_6P$ | 0.810612 | y = 9.1977x-1.1506 | 15 |
| **Glycine** | - | $C_2H_5NO_2$ | 0.992682 | y = 0.0017x-0.1506 | 0.0416 |
| **Histidine** | - | $C_6H_9N_3O_2$ | 0.587296 | y = 0.0017x-0.1506 | 0.0416 |
| **Isocitrat** | - | $C_6H_8O_7$ | 0.927715 | y = 0.0017x-0.1506 | 6.2 |
| **Isoleucine** | - | $C_6H_{13}NO_2$ | 0.986358 | y = 0.0077x-0.1506 | 0.2 |
| **Lactate** | - | $C_3H_6O_3$ | 0.988113 | y = 0.0007x-0.1506 | 0.0626 |
| **Leucine** | - | $C_6H_{13}NO_2$ | 0.972434 | y = 0.0127x-0.1506 | 0.104 |
| **Lysine** | - | $C_6H_{14}N_2O_2$ | 0.994367 | y = 0.5657x-5.1506 | 0.0416 |
| **Malat** | - | $C_4H_6O_5$ | 0.992823 | y = 0.0127x-0.1506 | 3.12 |
| **Methionine** | - | $C_5H_{11}NO_2S$ | 0.973717 | y = 0.0167x-0.1506 | 0.2 |
| **ortho-Tyrosine** | o-Tyrosine | $C_9H_{11}NO_3$ | 0.851967 | y = 0.0907x-0.1506 | 0.104 |
| **Phosphoenolpyruvate** | PEP | $C_3H_5O_6P$ | 0.999403 | y = 0.0007x-0.1506 | 0.62 |

| | | | | | |
|---|---|---|---|---|---|
| **Phenylalanine** | - | $C_9H_{11}NO_2$ | 0.982843 | y = 0.1867x-1.1506 | 0.0416 |
| **Proline** | - | $C_5H_9NO_2$ | 0.993064 | y = 0.7247x-7.1506 | 0.0416 |
| **Pyruvate** | - | $C_3H_4O_3$ | 0.999601 | y = 0.0057x-0.1506 | 0.3 |
| **Serine** | - | $C_3H_7NO_3$ | 0.980071 | y = 0.0277x-0.1506 | 0.0416 |
| **Succinate** | - | $C_4H_6O_4$ | 0.978143 | y = 0.0037x-0.1506 | 0.0626 |
| **Taurine** | - | $C_2H_7NO_3S$ | 0.981986 | y = 0.0077x-0.1506 | 0.0416 |
| **Threonine** | - | $C_4H_9NO_3$ | 0.992306 | y = 0.0427x-0.1506 | 0.0416 |
| **Tryptophan** | - | $C_{11}H_{12}N_2O_2$ | 0.948304 | y = 0.0520x-0.4891 | 0.4 |
| **Tyrosine** | - | $C_9H_{11}NO_3$ | 0.997303 | y = 0.0457x-0.1506 | 0.104 |
| **Valine** | - | $C_5H_{11}NO_2$ | 0.998356 | y = 0.2137x-2.1506 | 0.0416 |

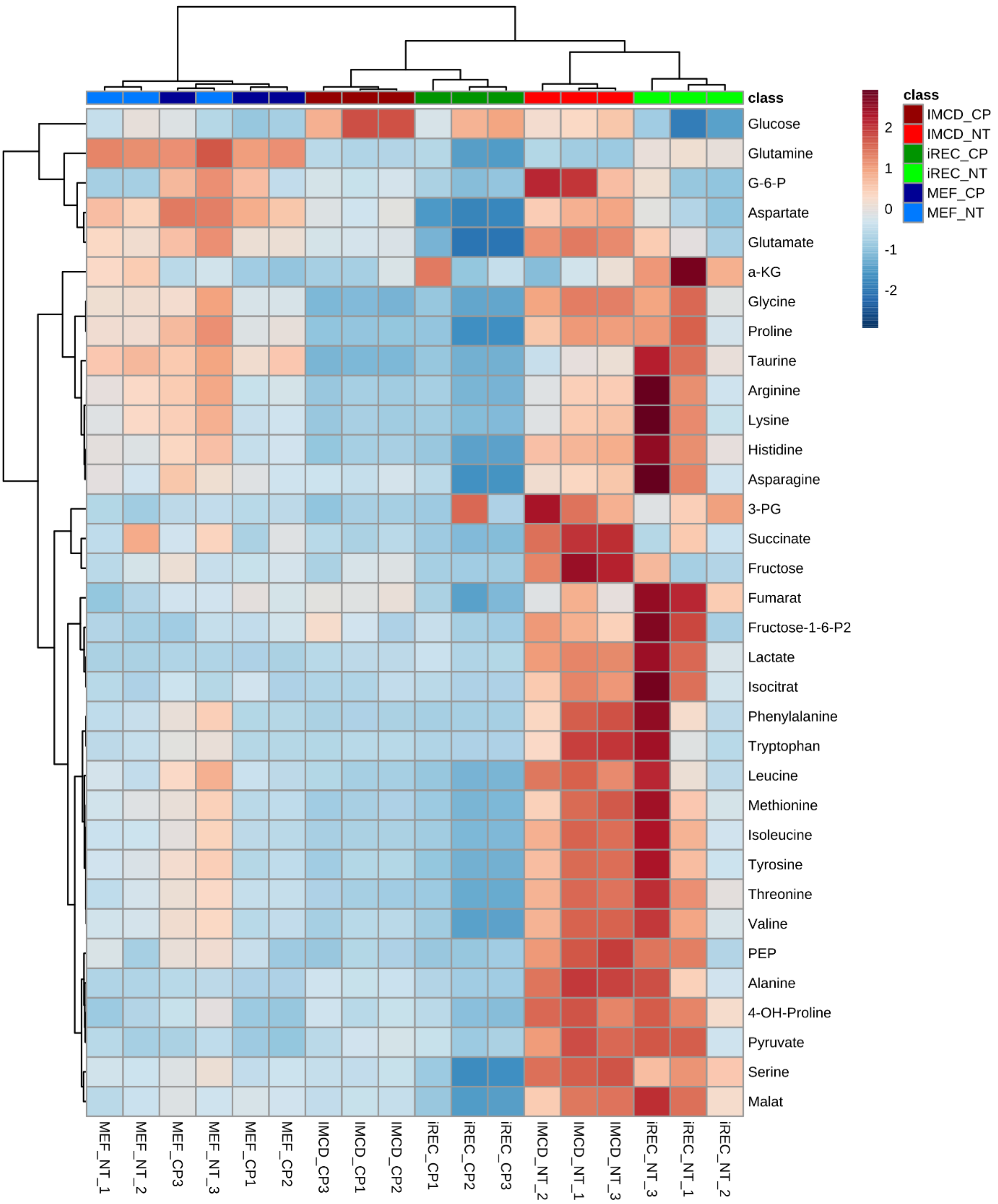


**Supplementary figure S1: Heat map and cluster analysis of endometabolome discriminates cisplatin treatment only in renal epithelial cells.** Range-scaled Z-scores of annotated features acquired by targeted multiple reaction monitoring (MRM). Displayed are metabolites with q-value < 0.05 according to ANOVA and FDR. No effect could be observed in MEFs after cisplatin application, while there was a strong influence on renal epithelial cells. Amino acids and intermediates of glycolysis and the TCA-cycle were concomitantly down-regulated by cisplatin in iRECs and mIMCD-3 cells, whereas glucose accumulated after cisplatin-treatment. Detection of phosphorylated glycolysis intermediates in cisplatin-treated samples were below the linear range. Therefore, these alterations should be judged qualitatively. Light color: untreated, dark color: Cisplatin-treated; blue: MEFs, red: mIMCD-3 cells, green: iRECs, each n=3

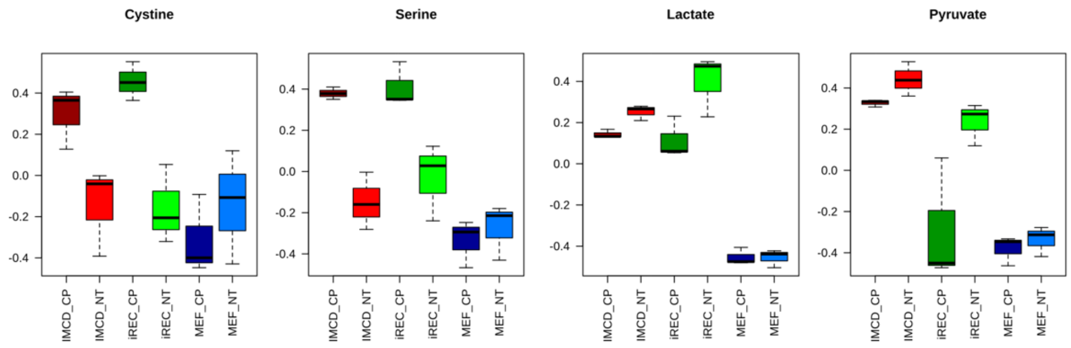


**Supplementary figure S2: Box plots of significantly altered exometabolites after cisplatin-treatment.** Range-scaled Z-scores of annotated features acquired by targeted MRM-acquisition. Exometabolites with a q-value < 0.05 according to ANOVA and FDR as well as a positive post-hoc test after treatment in the same cellular back-ground are shown. No alterations were observed in MEFs, serine was the sole metabolite significantly changed in mIMCD-3 cells and all displayed metabolites changed significantly in iRECs. Light color: untreated, dark color: Cisplatin-treated; blue: MEFs, red: mIMCD-3 cells, green: iRECs, each n=3
